# Harnessing A3G for efficient and selective C-to-T conversion at C-rich sequences

**DOI:** 10.1186/s12915-020-00879-0

**Published:** 2021-02-18

**Authors:** Wenxia Yu, Jianan Li, Shisheng Huang, Xiangyang Li, Ping Li, Guanglei Li, Aibin Liang, Tian Chi, Xingxu Huang

**Affiliations:** 1 School of Life Science and Technology, ShanghaiTech University, Shanghai, 201210 China; 2 University of Chinese Academy of Sciences, Beijing, 100049 China; 3 Shanghai Institute of Biochemistry and Cell Biology, Chinese Academy of Sciences, Shanghai, 200031 China; 4 Department of Hematology, Tongji Hospital of Tongji University, Shanghai, 200092 China; 5 Department Immunobiology, Yale University School of Medicine, New Haven, CT 06520 USA; 6 CAS Center for Excellence in Molecular Cell Science, Shanghai Institute of Biochemistry and Cell Biology, Chinese Academy of Sciences, Shanghai, 200031 China

**Keywords:** Base editing, Apobec 3G, Motif, C-rich

## Abstract

**Background:**

Site-specific C>T DNA base editing has been achieved by recruiting cytidine deaminases to the target C using catalytically impaired Cas proteins; the target C is typically located within 5-nt editing window specified by the guide RNAs. The prototypical cytidine base editor BE3, comprising rat APOBEC1 (rA1) fused to nCas9, can indiscriminately deaminate multiple C’s within the editing window and also create substantial off-target edits on the transcriptome. A powerful countermeasure for the DNA off-target editing is to replace rA1 with APOBEC proteins which selectively edit C’s in the context of specific motifs, as illustrated in eA3A-BE3 which targets TC. However, analogous editors selective for other motifs have not been described. In particular, it has been challenging to target a particular C in C-rich sequences. Here, we sought to confront this challenge and also to overcome the RNA off-target effects seen in BE3.

**Results:**

By replacing rA1 with an optimized human A3G (oA3G), we developed oA3G-BE3, which selectively targets CC and CCC and is also free of global off-target effects on the transcriptome. Furthermore, we created oA3G-BE4max, an upgraded version of oA3G-BE3 with robust on-target editing. Finally, we showed that oA3G-BE4max has negligible Cas9-independent off-target effects at the genome.

**Conclusions:**

oA3G-BE4max can edit C(C)C with high efficiency and selectivity, which complements eA3A-editors to broaden the collective editing scope of motif selective editors, thus filling a void in the base editing tool box.

## Background

C>T DNA base editors (CBEs), consisting of APOBEC proteins linked to nCas proteins, complement other forms of base editors (A>G base editors and the recently described prime editor), with great potential for basic research and disease treatment [1, 2]. However, the classic CBEs, namely BE3 comprising rat APOBEC1 (rA1) fused to nCas9, have several important limitations, including indiscriminate deamination of multiple cytidines in diverse editing motifs within the 5-bp editing window and massive off-target effects on the transcriptome. One approach to counter these undesirable activities is to mutate rA1, as illustrated in the BE3 variants named YE1-BE3 which bears W90Y/R126E [3, 4] and BE3-R33A/K34A [5]. Both variants display undetectable RNA editing and narrowed on-target editing window, the latter helping reduce editing at the bystander cytidines. However, the editing motifs of YE1-BE3 remain broad, and so the bystanders within the narrowed editing window remain susceptible to indiscriminate deamination by YE1. Remarkably, BE3-R33A/K34A preferentially targets the cytidines preceded by T (namely the cytidines in the TC motif, the target C underlined). This selectivity minimizes bystander editing but at the same time makes BE3-R33A/K34A largely inapplicable to CC, GC, or AC.

The second countermeasure is to exploit the natural diversity of APOBEC proteins. For example, the human APOBEC family comprises 11 members with diversified functional properties, including A3A which selectively edits TC [6] and A3G, which preferentially deaminates CCC as well as CC [7–10]. By replacing rA1 in BE3 with an engineered A3A, Joung and colleagues created eA3A-BE3 that preferentially edits TC, therefore minimizing the bystander editing as in BE3-R33A/K34A [11, 12]. However, just as BE3-R33A/K34A, eA3A-BE3 is inapplicable to CC, GC, or AC. It is thus highly desirable to develop editor targeting these three motifs.

Here, we present oA3G-BE3, which selectively edits CC and CCC and furthermore has a very narrow editing window and lacks detectable global off-target edits on RNA or DNA. We also describe oA3G-BE4max, which is as selective as oA3G-BE3 but more active. These novel editors complement eA3A-BE3 and BE3-R33A/K34A to broaden the collective editing scope of highly selective editors.

## Results

### Development of oA3G-BE3

**Fig. 1:**
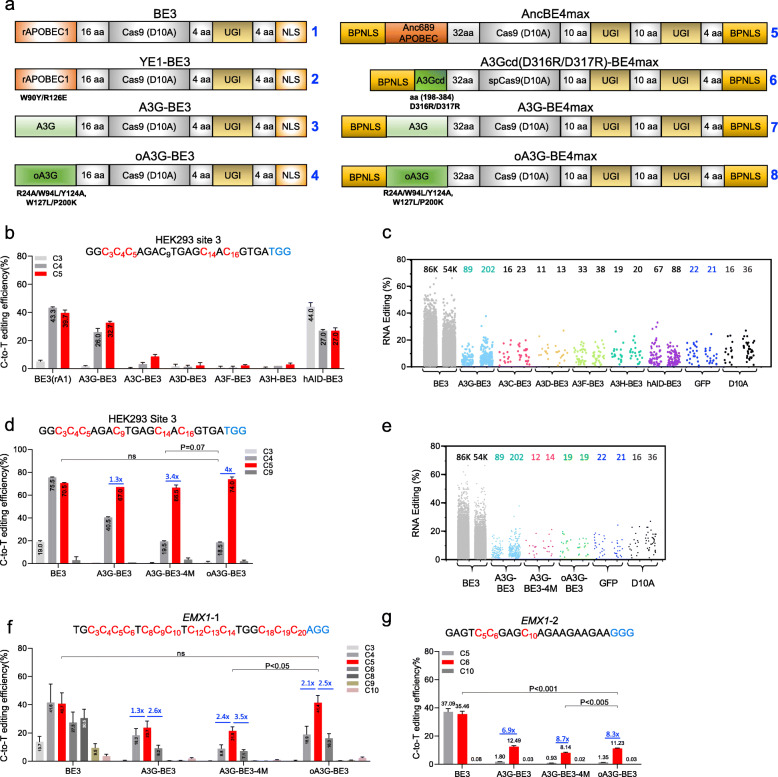
The development of oA3G-BE3. **a** Key editors tested in this study. Editors #1 [13], #2 [3, 4] #3 [26], #5 [14], and #7 [15] have been formally published whereas #6 posted in a preprint [16]. In contrast, editors #4 (oA3G-BE3) and #8 (oA3G-BE4max) are developed in this study, where oA3G denotes “optimized A3G” bearing 5 substitutions. The BE4max architecture differs from that of BE3 in that it has 2 copies of UGI, optimized codon usage, and nuclear localization, in addition to using optimized linker sequences between the fused proteins. NLS, nuclear localization signal; BPNLS, bipartite nuclear localization signal; UGI, uracil DNA glycosylase inhibitor. **b** A3G-BE3 efficiently edited CCC present at HEK293 site 3. Various editors and a gRNA for the target site were coexpressed in HEK293T cell and analyzed using Sanger sequencing. The target sequence is depicted. The third C at CCC was edited most efficiently by A3G-BE3 and highlighted in the bar graph. Values are mean ± SEM from triplicate transfections. **c** A3G-BE3 created low levels of off-target edits at the transcriptome. Editors were coexpressed with the gRNA targeting HEK293 site 3 as in **a**, but transfection was done in duplicates and at a larger scale. Cells with top 15% GFP signal were sorted and analyzed by RNA-seq 48 h later. The jitter plot shows the off-target edits in duplicate samples, with the total numbers of the edits indicated. **d**, **e** On-target (**d**) and RNA off-target (**e**) editing by A3G-BE3-4M and oA3G-BE3. The samples are from the same experiment as in **b**, but the cells with top 15% GFP signal were analyzed in parallel by Sanger sequencing (to measure on-target editing at HEK293 site 3; **d**) and RNA-seq (to determine RNA off-target effects; **e**). The on-target editing rates were higher than **b** because the cells analyzed here, with top 15% GFP fluorescence, expressed higher levels of editors and gRNA. The bar graph in **d** displays mean ± SEM from duplicate transfections, with the blue numbers being the ratios of the editing rates at the target C (C_5_, red bar) over that at the bystander (C_4_), which is a measure of the selectivity of the editors. The *P* value (0.07%) for the on-target editing rates has not reached significance presumably due to the small sample size (*n* = 2). **f**, **g** On-target editing by A3G-BE3-4M and oA3G-BE3 at two more sites. Editors and gRNAs were coexpressed as in **a**. Gene editing was then analyzed by targeted deep-sequencing instead of Sanger sequencing, in order to detect the low level editing at C_5_ at *EMX1*-2

A3G has a duplicated deaminase domain structure, with the C-terminal domain catalyzing cytidine deamination while the N-terminal domain has poorly understood regulatory functions [6]. We opted to use the entire protein for base editing. To harness A3G, we replaced the rA1 in BE3 with human hA3G (Fig. 1a, editors #1 and #3) and assayed its on-target editing in HEK293T cells at a well-defined genomic site carrying CCC within the editing window (HEK293 site 3). We found that A3G-BE3 was indeed capable of editing both the second C and the third C at CCC, but with the third C edited preferentially as expected (26% vs. 32%; Fig. 1b); hereafter, the preferentially edited C’s within the editing window will be considered the target C’s and the remaining the bystanders. Not surprisingly, A3G-BE3 outperformed alternative CBEs comprising BE3 fused to other members of APOBEC family tested (A3C, A3D, A3F, A3H, AID) in terms of editing efficiency or selectivity (Fig. 1b). However, A3G-BE3 seemed somewhat weaker than BE3. Furthermore, although the off-target edits created by A3G-BE3 at the transcriptome were dramatically reduced compared with that created by BE3 (89–202 vs. 54,469–85,917), they remained clearly above the background (< 35 edits, as seen in cells expressing GFP or nCas9; Fig. 1c). Accordingly, we took two steps to optimize A3G-BE3.

First, we sought to eliminate the RNA editing. The N-terminal domain (aa 1–196) can bind RNA, and four residues (R24, W94, Y124, and W127) have each been clearly shown to be important for association with multiple RNA targets through mutagenesis and structural studies [18–22]. To ensure complete elimination of binding to any RNA, we mutated all the 4 residues (R24A, W94L, Y124A, and W127L) and found that indeed, the resulting mutant A3G-BE3-4M created only 12–14 edits, comparable to GFP (21–22) or nCas9 (16–36; Fig. 1d). The loss of off-target edits was not an artifact resulting from nonspecific inactivation of A3G-BE3-4M, as the mutant edited the target C at HEK293 site 3 as efficiently as A3G-BE3 (Fig. 1d). Unexpectedly, the mutations also increased the selectivity of A3G-BE3 for the target C relative to the bystander. Specifically, A3G-BE3 displayed only a slight (1.3×) preference for the target C over the preceding C (67% vs. 41% editing rates), but the preference was much stronger (3.4×) for A3G-BE3-4M (67% vs. 20%; Fig. 1d). In other words, the mutations inhibited off-target editing not only at RNA but also at the bystander C within the editing window, but remarkably, on-target editing was not compromised, at least at the CCC at HEK293 site 3.

We next extended the analysis by testing the editors at two more motifs, namely CC and CCCC, present at *EMX1*-2 and *EMX1*-1, respectively. A3G-BE3 edited CC with a 6.9× preference over the bystander, but A3G-BE3-4M showed stronger (8.7×) preference (Fig. 1f), and the same trend was seen at CCCC, where the preference of the target C over the two flanking bystanders was 1.3×–2.6× for A3G-BE3 but 2.4×–3.5× for A3G-BE3-4M (Fig. 1g). These data reinforce the notion that the quadruple mutations improved the editor specificity not only at RNA but also within the editing window on DNA. The mutagenesis approach was thus successful. Of note, since our focus was on method development rather than mechanistic understanding, we have not sought to dissect the contributions of the individual mutations to the performance of A3G-BE3-4M.

Our second step of optimization was aimed at increasing the editing efficiency of A3G-BE3-4M. A3G-BE3-4M, just as A3G-BE3, was less efficient than BE3, the editing rates at the target C’s being 35%, 12%, and 8% for BE3, A3G-BE3, and A3G-BE3-4M respectively at *EMX1*-2 (Fig. 1f), and 41%, 24%, and 22% for the three editors at *EMX1*-1 (Fig. 1g). To potentiate A3G-BE3-4M, we singularly mutated multiple residues in A3G-BE3-4M that are (potentially) capable of impacting editing rates, including D128, P198, P200, and Q322. Specifically, P199A, P200A, and Q322K are present in a previously engineered A3G variant with enhanced catalytic activity, whereas D128 is located near the A3G dimer interface [21], and we speculated that D128K might also alter A3G catalytic activity. Finally, we also tested P199W and P200K. We compared all these mutants at a total of 5 target sites, including 3 sites described in Fig. 1. All these mutations (largely) failed to potentiate A3G-BE3-4M except P200K (Fig. S1), and consequently, the optimized editor bearing the 5 mutations (R24A, W94L, Y124A, W127L, P200K) was named “optimized A3G-BE3” or “oA3G-BE3” (Fig. 1a, #4). oA3G-BE3 displayed higher on-target editing rates than A3G-BE3-4M at all three sites shown in Fig. 1, particularly at *EMX1*-1 where the efficiency was increased from 22 to 41%, namely to a level identical to that achieved by BE3 (Fig. 1g). The increases in the efficiency were less obvious at the other two sites, but still statistically significant (*P* < 0.005, Fig. 1f) or nearly so (*P* = 0.07, Fig. 1d). The same trend was seen at the other 5 sites tested (Additional file 1: Fig. S1).

Collectively, these data indicate that oA3G-BE3 is more specific, more selective, and more active than A3G-BE3.

### oA3G-BE3 outperforms YE1-BE3 at C-rich motifs

Although oA3G-BE3 is presumably the top choice for selective editing at C-rich motifs, YE1-BE3 is a strong rival given its two outstanding features: reduced editing window width and lack of off-target effects on RNA or DNA [3, 4, 15]. We thus compared the two editors side by side, together with their common predecessor BE3, at the panel of 8 sites described in Additional file 1: Fig. S1. These sites carry increasing numbers of C, from the simple CC (*EMX1*-2) to the complex CCCCCCC (*DNMT3B*-2).

**Fig. 2:**
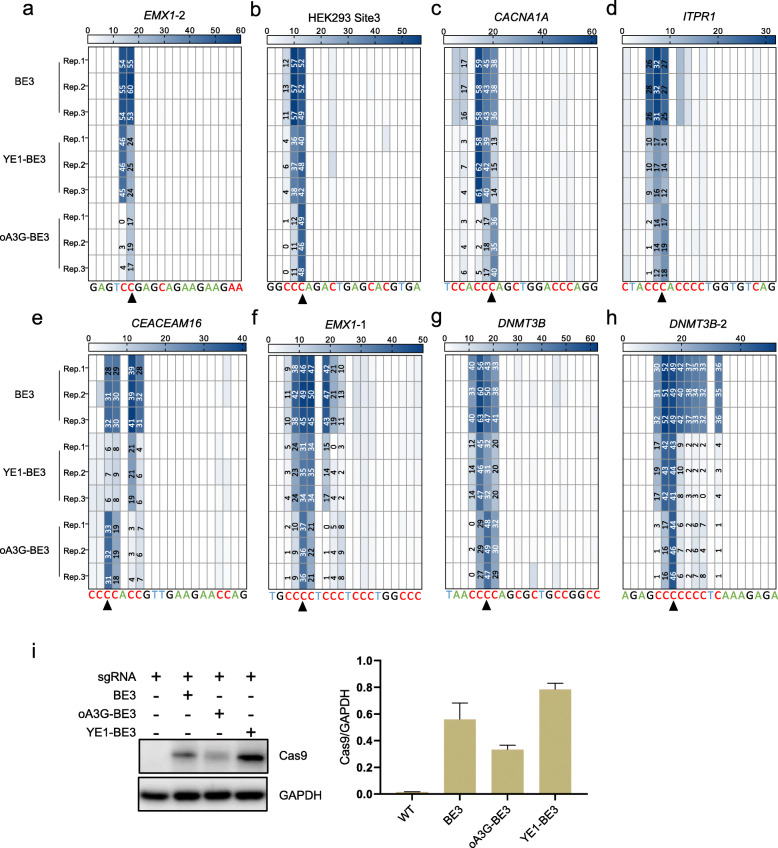
Benchmarking oA3G-BE3 against YE1-BE3 at diverse targets. Heatmaps showing editing rates at 8 target sites, including the three used in Fig. 1 (**a**–**h**), and Western blot measuring editor expression (**i**). These sites collectively present editing motifs with a wide range of complexity. *EMX1*-1 and *EMX1*-2 are two adjacent sites on the same gene. *CACNA1A* and *ITPR1* have naturally occurring pathogenic C>T mutations at the indicated C. Arrows indicate the intended target C for oA3G-BE3. Data are from triplicate transfections

We first examined editing at the CC motif present at *EMX1*-2 (Fig. 2a). For convenience, we will refer to the motif as C_1_C_2_. Consistent with Fig. 1f, oA3G-BE3 could edit C_2_ (18%) but not C_1_ (< 3%), whereas BE3 edited the two C’s with nearly identical efficiencies (~ 56%). YE1-BE3 proved more selective than BE3, but the selectivity was dramatically lower than oA3G-BE3, as it edited the two C’s with less than a 2× difference in the efficiency (46% vs. 26%), Thus, at CC, oA3G-BE3 was far more selective than YE1-BE3, let alone BE3 (Fig. 2a).

We then compared the editors at C_1_C_2_C_3_ (Fig. 2b–d). The scenario here is more challenging, as C_2_ is susceptible to deamination by oA3G-BE3, contrary to the counterpart at CC. Nevertheless, oA3G-BE3 again outperformed YE1-BE3 at all the C_1_C_2_C_3_ sites tested (HEK293 site 3, *CACNA1A*, *ITPR1*). Specifically, while oA3G-BE3 indeed edited C_2_, C_3_ was edited more efficiently, the rates exceeding C_2_ by 4×, 2×, and 1.4×, respectively, at the three sites (48% vs. 11% at HEK293 site 3, 37% vs.17% at *CACNA1A*, and 18% vs. 13% at *ITPR1*; Fig. 2b–d). In contrast, YE1-BE3 proved much less discriminative: at HEK293 site 3, C_2_ and C_3_ were edited with similar efficiencies; at *ITPR1*, all three C’s were almost equally edited; and finally, at *CACNA1A*, the three C’s were also all substantially edited, although to different extents (14% to 60%). It is noteworthy that the C_3_>T mutations at *CACNA1A* and *ITPR1* (ClinVar accession # VCV000008504.1 and RCV000015924.27, respectively) are both pathogenic, illustrating the ability of oA3G-BE3 to install clinically relevant mutations.

Next, we examined editing at even more challenging motifs, namely at C_1_C_2_C_3_ followed by 1 C (*CEAEAM16* and *DNMT3B*), 2 C’s (*EMX1*-1), or 4 C’s (*DNMT3B*-2; Fig. 2e–h). Although oA3G-BE3 could edit all the C’s except C_1_, C_3_ was invariably the most efficiently edited at all the 4 sites, whereas YE1-BE3 again invariably proved less discriminative. For example, at *CEAEAM16* (Fig. 2e), whereas oA3G-BE3 showed a 1.7× preference for C_3_ relative to C_4_ (32% vs. 19%), the two C’s were edited with similar frequencies by YE1-BE3 (~ 8%). Thus, oA3G-BE3 edited the target (C_3_) more efficiently and more selectively than YE1-BE3 at C_1_C_2_C_3_C_4_ within the *CEAEAM16* site. Note that within the editing window at the *CEAEAM16* site, a CC dinucleotide is present downstream of C_1_C_2_C_3_C_4_, and YE1-BE3 (but not oA3G-BE3) was able to edit a C efficiently (20%), reinforcing the notion about superior selectivity of oA3G-BE3 relative to YE1-BE3 (Fig. 2e).

The C-rich motifs at *CEAEAM16*, *DNMT3B*, *EMX1*-1, and *DNMT3B*-2 enabled us to estimate the “editing window width,” a key attribute of DNA base editors defined as “the number of nucleotide positions at a given site for which editing efficiency exceeds the half-maximal value for that target site” [3]. For oA3G-BE3, the width at *CEAEAM16*, *DNMT3B*, *EMX1*-1, and *DNMT3B*-2 was 2, 3, 2, and 1 nt, respectively, which is similar to that of YE1-BE3 (1, 2, 3, 2) but narrowed than BE3 (5, 5, 4, 9). Thus, the editing window of oA3G-BE3, as narrow as that of YE1-BE3, is one of the narrowest among all CBE variants.

Another key attribute of DNA base editors is the “activity window,” which is the region of DNA, defined by the number of nucleotides from PAM, where a base editor can induce efficient point mutations [2]. Based on the performance of oA3G-BE3 and A3G-BE3 at the 8 sites tested, the activity windows for both editors should span C3–C7, which is consistent with the fact that the activity window for most base editors is approximately four to five nucleotides wide [2].

These data indicate that oA3G-BE3 but not YE1-BE3 can edit C(C)C with high selectivity. The same conclusion was reached regardless of the doses of plasmids transfected (Additional file 2: Fig. S2a), and when the editors were tested in a different human cell line than HEK293 (Additional file 3: Fig. S3), indicating that the high selectivity of oA3G-BE3 is an intrinsic property of the editor. Of note, our data also indicate that the editing efficiency of oA3G-BE3 was similar to YE1-BE3 but lower than BE3, as judged from the editing rates at the targeted C’s. Interestingly, Western blot analysis revealed that oA3G-BE3 was expressed at markedly lower levels than YE1-BE3 and BE3, suggesting that the intrinsic efficiency of oA3G-BE3 could be much higher (Fig. 2i). Finally, we have also analyzed BE3-R33A/K34A, the TC-selective CBE lacking RNA off-target effects mentioned before, confirming that the editor was inapplicable to CCC (not shown) [5].

### oA3G-BE4max outperforms AncBE4max and A3Gcd(D316R/D317R)-BE4max in target selectivity

**Fig. 3:**
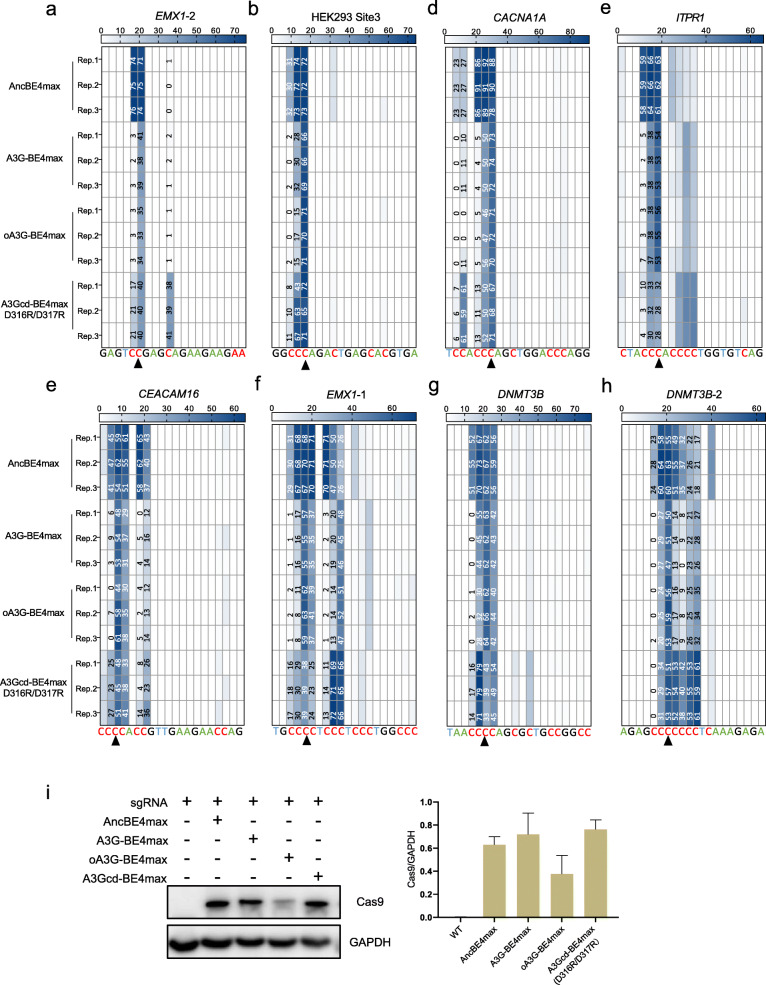
oA3G-BE4max benchmarked against alternative editors. The experiments were the same as that shown in Fig. 2 except for the editors used

Compared with BE3, the 4th generation CBE named AncBE4max is substantially more active, representing the state of the art in C>T editing [14]. We thus created oA3G-BE4max and benchmarked it against AncBE4max as well as A3G-BE4max [15] (Fig. 1a, #8, 5, 7, respectively). oA3G-BE4max proved somewhat less active but far more selective than AncBE4 at all the 8 target sites tested, and slightly more active and selective than A3G-BE4max (Fig. 3) as in the case of oA3G-BE3 vs. A3G-BE3 (Fig. 2). On the other hand, oA3G-BE4max was as selective as oA3G-BE3 (compare Fig. 3 with Fig. 2), but should be more active than oA3G-BE3 as inferred from the known properties of BE4max and BE3. We thus recommend oA3G-BE4max for efficient and selective editing at C(C)C.

We noted that A3G-BE4max derivative has been described in a preprint [16], subsequently published during the review of the current paper [17] where rA1 in BE4max was replaced with the C-terminal catalytic domain (cd) of A3G to create A3Gcd-BE4max that is more selective than BE4max, preferentially deaminating cytidines according to a CCC ≥ CCC ≥ CC hierarchy. A3Gcd-BE4max, however, is far less active than BE4max, which can be remedied to some extent by engineering D316R/D317R into A3Gcd [16]. A3Gcd(D316R/D317R)-BE4max (Fig. 1a, #6) was indeed more selective than BE4max at some of the 8 sites we tested, but far less selective than oA3G-BE4max at all the 8 sites (Fig. 3). Interestingly, the preferentially edited C’s in C-rich motifs also differed between oA3G-BE4max and A3Gcd(D316R/D317R)-BE4max. Thus, while oA3G-BE4max (and oA3G-BE3 as well) preferred the third C’s of the first CCC motif within the editing window at all the targets examined, A3Gcd(D316R/D317R)-BE4max did not show any consistent preference at different targets. Thus, the N-terminal domain in oA3G may influence the selection of the target C by the C-terminal domain.

The results above collectively demonstrate that oA3G-BE4max is more selective than AncBE4max and A3Gcd(D316R/D317R)-BE4max in HEK293T cells. A similar trend was seen in a different cell line (Additional file 3: Fig. S3). We have also compared editor expression by Western blot, finding oA3G-BE4max the least abundant, suggesting higher intrinsic activity of oA3G-BE4max as in the case of oA3G-BE3 (Fig. 3i).

### Cas9-independent deamination in HEK293T cells: orthogonal R-loop assay

**Fig. 4:**
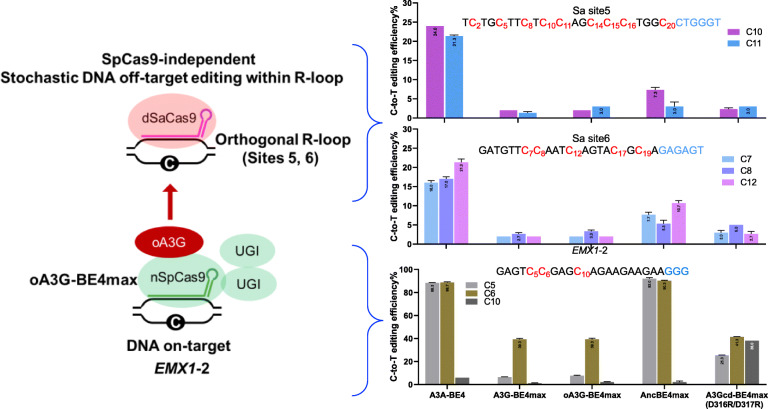
Cas9-independent editing on DNA. The principle of the assay is illustrated at the left using oA3G-BE4max as an example, whereas the results are shown as bar graphs at the right. dSaCas9 recruited by a gRNA generates an R-loop at the specified genomic site (sites 5 and 6 in the current study; top left). A subset of the C’s within the R-loop is potentially susceptible to stochastic deamination by oA3G carried in oA3G-BE4max. On-target editing (at the *EMX1*-2) is induced to serve as a positive control for the off-target editing (bottom left). dSaCas9 and its gRNAs targeting site 5 or site 6 were coexpressed in HEK293T cells with the indicated editors and the gRNA targeting *EMX1*-2, and the editing analyzed 3 days later by Sanger sequencing. For on-target editing, only the data that controlled for site 5 editing is shown; the data for site 6 is highly similar and omitted for clarity. Values in the bar graphs are mean ± SEM (*n* = 3). Sanger chromatograms are shown in Additional file 4: Fig. S4

CBEs can potentially create stochastic Cas9-independent off-target edits on the genome, typically at frequencies well below the ~ 0.1% detection limit of practical high-throughput DNA sequencing experiments [15]. Exploiting the fact that the deaminases used in CBEs can only act on ssDNA, a fast, sensitive, and cost-effective assay (“orthogonal R-loop assay”) has recently been described to detect such rare events, in which dSaCas9 together with a gRNA are used to induce a stable, ssDNA region (orthogonal R loop) at specific locus, thus artificially magnifying Cas9-independent deamination (Fig. 4, left) [15]. Using this assay, Doman et al. measured Cas9-independent edits on DNA for over a dozen CBEs including A3G-BE4max [15]. A3G-BE4max proves one of the most specific CBEs available: in HEK293T cells, the off-target editing rates at 18 cytidines at 6 orthogonal R-loops averaged only ~ 0.3%, even lower than YE1 (~ 0.8%), a classic high-specificity mutant (Fig. 3a in Doman et al.’s paper). We therefore used the R-loop assay to assess the Cas9-independent off-target effect of oA3G-BE4max at sites 5 and 6, the most sensitive sites for detecting off-target effects for diverse editors [15]. As reported, at both sites, A3A-BE4 displayed high-level (> 20%) whereas A3G-BE4max little or no (< 5%) editing (Fig. 4, top two bar graphs; see Additional file 4: Fig. S4 for raw data). Importantly, oA3G-BE4max behaved similarly to A3G-BE4max, with little or no editing, which is perhaps not surprising. Interestingly, although AncBE4max editing was clearly detectable, it seemed less active than BE4max described previously [15]. Finally, A3Gcd(D316R/D317R)-BE4max was also highly specific.

These data suggest that oA3G-BE4max, and by inference, oA3G-BE3, did not induce detectable Cas9-independent editing at the genome.

## Discussion

By exploiting the natural substrate selectivity of human A3G and improving it via introduction of 5 substitutions, we have created oA3G-BE3, the first CBE optimized for highly selective cytidine deamination at CC and CCC, with the off-target effects undetectable on either DNA or RNA. We have also created oA3G-BE4max with boosted editing efficiency relative to oA3G-BE3. oA3G-BE3/BE4max can thus complement eA3A- and R33A/K34A-editors to expand the editing scopes for motif-selective editors. But there is a room for improvement for eA3A-BE3/BE4max. First, although the editors can deaminate CCC with high efficiency, the editing rates at CC remain moderate, which could perhaps be addressed partly by optimizing eA3A codon usage. In addition, the activity windows of the editors might be widened using circularly permutated Cas9 variants [23], and the scope of the targetable sites broadened using the near-PAMless Cas9 variant [24].

The oA3G-editors should be highly useful for rescuing and installing disease-relevant mutations. Specifically, the ClinVar database has documented 1649 and 4018 pathogenic C-to-T SNVs in the contexts of NCCCN and DCCD motifs, respectively, where N is any nucleotide, D is A/G/T, and the underlined is the C mutated in the patients. oA3G-editors should be useful for installing these mutations. On the other hand, there are 750 and 950 pathogenic T-to-C SNVs in the contexts of NCCTN and DCTD, respectively, where the T is mutated to C in the patients, and oA3G-editors should be useful for correcting these mutations.

Among the 5 substitutions carried in oA3G, R24A, W94L, Y124A, and W127L eliminated off-target effects on RNA and increased the selectivity on target C within the editing window, while P200K boosted the on-target editing efficiency on DNA. The mechanisms of action of these mutations are not entirely clear, partly due to the lack of a co-crystal structure of hA3G in complex with RNA. However, rhesus macaque A3G (rA3G) is highly similar to the human protein, with all the 5 residues mutated in oA3G conserved except P200, and rA3G structure has been solved, which provides some clues regarding the elimination of RNA off-target editing [22] (Additional file 5: Fig. S5). Specifically, rA3G is dimeric in solution, with 18 residues (including R24, Y124, and W127) aligned around the dimer junction in a way suitable for binding RNA. Through dimerization, the R24 and a few positive residues nearby in each monomer are brought into close proximity, which markedly enhances the local positive electrostatic potentials (PEP). Thus, dimerization promotes RNA binding, and the latter in turn might stabilize dimerization via a positive feedback loop [22]. According to this model, mutations in R24, Y124, and W127 might disrupt RNA contact with the hA3G dimer, leading to dimer dissociation, which would in turn further impair RNA binding. Indeed, mutagenesis experiments show that R24A, Y124A, and W127L each impair hA3G oligomerization and RNA association, and W94L has similar effects [18–20]. However, W94 is not among the 18 residues at the dimer junction, and so how/whether W94L impairs RNA binding is unclear. Neither is it clear how the quadruple mutations increase the selectivity of A3G-BE3 for the target C relative to the bystander. Finally, it is also unclear how exactly P200K works, but it might act by somehow increasing hA3G catalytic activity as reported [25].

To our knowledge, base editors using A3G have been described in two formal publications. First, as mentioned above, Doman et al. find A3G-BE4max one of the most specific CBEs available [15]. Second, Martin et al. developed a panel of GFP reporters carrying the TCA editing motif and used them to compare the editing activities of 7 human APOBEC3 enzymes including A3G, finding A3G-BE3 unable to edit the C, as expected from the property of A3G [30]. Neither study is focused on A3G, and so neither addresses the crucial issues regarding the A3G-editors, such as on-target editing of C-rich sites and off-target effects on the transcriptome. The third base editor using A3G is A3Gcd(D316R/D317R)-BE4max, described in the preprint [16]. However, the global off-target effects on the transcriptome or genome were not analyzed in the study. In any case, we have found A3Gcd(D316R/D317R)-BE4max far less selective than oA3G-BE4max, which might result from its lack of the N-terminal domain of A3G.

## Conclusion

We have developed oA3G-BE4max for efficient and selective editing of C(C)C, which complements eA3A- and rA1(R33A/K34A)-editors to broaden the collective editing scope of motif selective editors.

## Methods

### Plasmids

gRNA and editor expression vectors were constructed using standard methods [26], as detailed in Supplemental Information. Key plasmids will be deposited at Addgene.

### Cell culture and transfection

The human embryonic kidney HEK293T cells (ATCC) were cultured at 37 °C with 5% CO_2_ in Dulbecco’s modified Eagle’s medium (DMEM) (10566, Gibco/Thermo Fisher Scientific) containing high glucose, sodium pyruvate, penicillin-streptomycin, and 10% fetal bovine serum (Gemini). Cells were passaged 3 times per week and tested to exclude mycoplasma contamination. For transfection, cells were seeded at proper density into 24-well or 6-well plates so that they reached 70% confluency the following day. Transfections were performed with Lipofectamine 3000 per the manufacturer’s instruction. Briefly, DNA was mixed with 2 μl Lipofectamine P3000 (Thermo Fisher Scientific, L3000015) and 25 μl Opti-MEM (Invitrogen) and incubated for 5 min at room temperature. 1.5 μl of the Lipofectamine 3000 (Thermo Fisher Scientific, L3000015) was diluted into 25 μl Opti-MEM (Invitrogen) and combined with the DNA:P3000 mixture, incubated for another 15 min at room temperature. The DNA:P3000:Lipofectamine 3000 mixture was added dropwise into the 24-wells.

### Analysis of on-target editing

Vectors expressing base editors (628 ng) and gRNA-puromycin resistance gene (373 ng) were co-transfected into HEK293T cells in 24-well plates (JETBIOFIL). Puromycin (InvivoGen) was added 24 h later to a final concentration of 2 μg/ml. Cells were harvested 72 h after transfection and genomic DNA extracted using QuickExtract™ DNA Extraction Solution (Lucigen). The editing rates were determined by Sanger sequencing except for Fig. 1d, e where deep-seq was used instead mainly for detecting editing at the bystander at *EMX1*-2. The results obtained with the two sequencing methods are in good agreement except when the editing rates fall below 10%, where only deep-seq remains reliable. Thus, we routinely used Sanger sequencing unless higher sensitivity is needed as in Fig. 1e. For Sanger sequencing, the target sites (~ 300 bp) were amplified using Phanta® Max Super-Fidelity DNA Polymerase (Vazyme) in a touchdown PCR with the following parameter: 94 °C 5′, followed by 10 cycles of 94 °C 30″, 68 °C (− 1 °C/cycle) 30″, and 72 °C 30″, followed by 15 cycles of 94 °C 30″, 58 °C 30″, and 72 °C 30″. The sequencing chromatograms were analyzed using EditR, an “accurate, fast, and low-cost method for the identification and quantification of base editing from fluorescent Sanger sequencing data” [27, 28]. The primer sequences are provided in Supplemental Information. For deep-seq, the same target regions were amplified in two rounds of PCR using Phanta® Max Super-Fidelity DNA Polymerase (Vazyme) to add Illumina adaptors and sample barcodes. The amplicons were then sequenced using Illumina Nextseq 500 (2 × 150 PE). BWA and Samtools were employed for mapping the pair-end reads to human reference genome (hg38), and VarDict for calling single nucleotide variants (SNVs) in the amplicon aware mode. The aligned reads were visualized using the Integrated Genome Viewer (IGV) and tabbed using Pysamstats. Primers used for sequencing are listed in Additional file 6.

### Analysis of Cas9-independent editing at the genome

Orthogonal R-loop assay was performed as described [15]. Briefly, to check off-target editing at site 5, plasmids expressing the dSaCas9 (300 ng) and its gRNA targeting site 5 (200 ng) were co-transfected into HEK293T cells with plasmids expressing an indicated editor (300 ng) and its gRNA targeting *EMX1*-2 (200 ng). Cells were treated with puromycin, and the editing at both site 5 and *EMX1*-2 detected by Sanger sequencing as described in the previous section. Off-targeting at site 6 was examined in the same way, except that the plasmid expressing the sgRNA for site 6 was used instead of the site 5 gRNA.

### Western blot

Vectors expressing the editors (4 μg) and gRNA-puro (2 μg) were co-transfected into HEK293T cells in 6 cm dish (JETBIOFIL). Puromycin (InvivoGen) was added 24 h later to a final concentration of 2 μg/ml. Cells were harvested 72 h after transfection, and total protein extracted by RIPA lysis (EpiZyme). The protein samples were separated by SDS-PAGE, transferred to PVDF membrane (Merck Millipore). After blocking with 5% (w/v) non-fat milk dissolved in TBST (25 Mm Tris, PH 8.0, 150 Mm NaCl, and 0.1% Tween 20) for 1 h, the membranes were incubated overnight with anti-CRISPR-Cas9 antibody (Abcam # ab204448) or anti-GAPDH antibody (Absin #abs132004) at 4 °C. After extensive washing, the membranes were incubated with HRP-conjugated secondary antibodies at room temperature for 1 h. Proteins were visualized using Enhanced Chemiluminescence (ELC) reagent (Merck Millipore) and detected with an Amersham Imager 600.

### Analysis of RNA off-targets

Vectors expressing the editors or nCas9 (4 μg) and gRNA-GFP (2 μg) were co-transfected into HEK293T cells in 6 cm dish (JETBIOFIL). Cells with top 15% GFP signal were harvested using FACS 48 h later, and total RNA extracted using the TRIzol reagent (Vazyme). The mRNA fraction was then enriched using a NEBNext Poly(A) mRNA Magnetic Isolation Module (NEB) before library construction using NEBNext Ultra RNA Library Prep Kit for Illumina (NEB). The libraries were sequenced on an Illumina HiseqXten-PE150, at a depth of around 20 million reads per sample. The reads were mapped to the human reference genome (hg38) by STAR software (version 2.5.1), with annotations from GENCODE version v30. After removing duplications and subtracting the reads in non-transfected cells, variants were identified by GATK (version 4.1.2) Mutect2 and filtered with FilterMutectCalls. The depth for a given edit should be at least 10×, and these edits are required to have at least 99% of reads supporting the reference allele in the wild-type samples. Finally, only C-to-T edits in transcribed strand are considered for downstream analysis.

### Statistics

Statistical significance throughout the paper was calculated using two-tailed Student’s *t* test, and data represented as mean ± SEM.

## Supplementary information

**Additional file 1: Fig. S1.** oA3G-BE3 outperforms the A3G-BE3 derivatives bearing different A3G mutations.

**Additional file 2: Fig. S2.** Effects of transfection condition on editing by BE3-editors.

**Additional file 3: Fig. S3.** Editor performance in U2OS cells.

**Additional file 4: Fig. S4.** Sanger chromatograms for off-target editing.

**Additional file 5: Fig. S5.** Potential mechanisms of action of the point mutations in oA3G.

**Additional file 6.** 1. sgRNA expression vectors. 2. Base editor expression vectors. 3. Primers used for detecting editing at the genomic DNA.

**Additional file 7.** Source data of Figure.

**Additional file 8.** Source data of SupFigure.

## Data Availability

All data generated or analyzed during this study are included in this published article and its supplementary information files (Additional file 1, 2, 3, 4, 5, 6, 7, 8). The RNA-seq and deep-seq data are available in the [NCBI Bioproject] repository (https://www.ncbi.nlm.nih.gov/bioproject/PRJNA642262) [29].
