# Supplementary material for: Harnessing A3G for efficient and selective C-to-T conversion at C-rich sequences
Source: BMC Biol. 2021 Feb 18;19:34. doi: 10.1186/s12915-020-00879-0 (PMC7893952; doi:10.1186/s12915-020-00879-0)
Supplement: Supplementary file 1 — Additional file 1: Fig. S1. oA3G-BE3 outperforms the A3G-BE3 derivatives bearing different A3G mutations. [file 12915_2020_879_MOESM1_ESM.pdf]

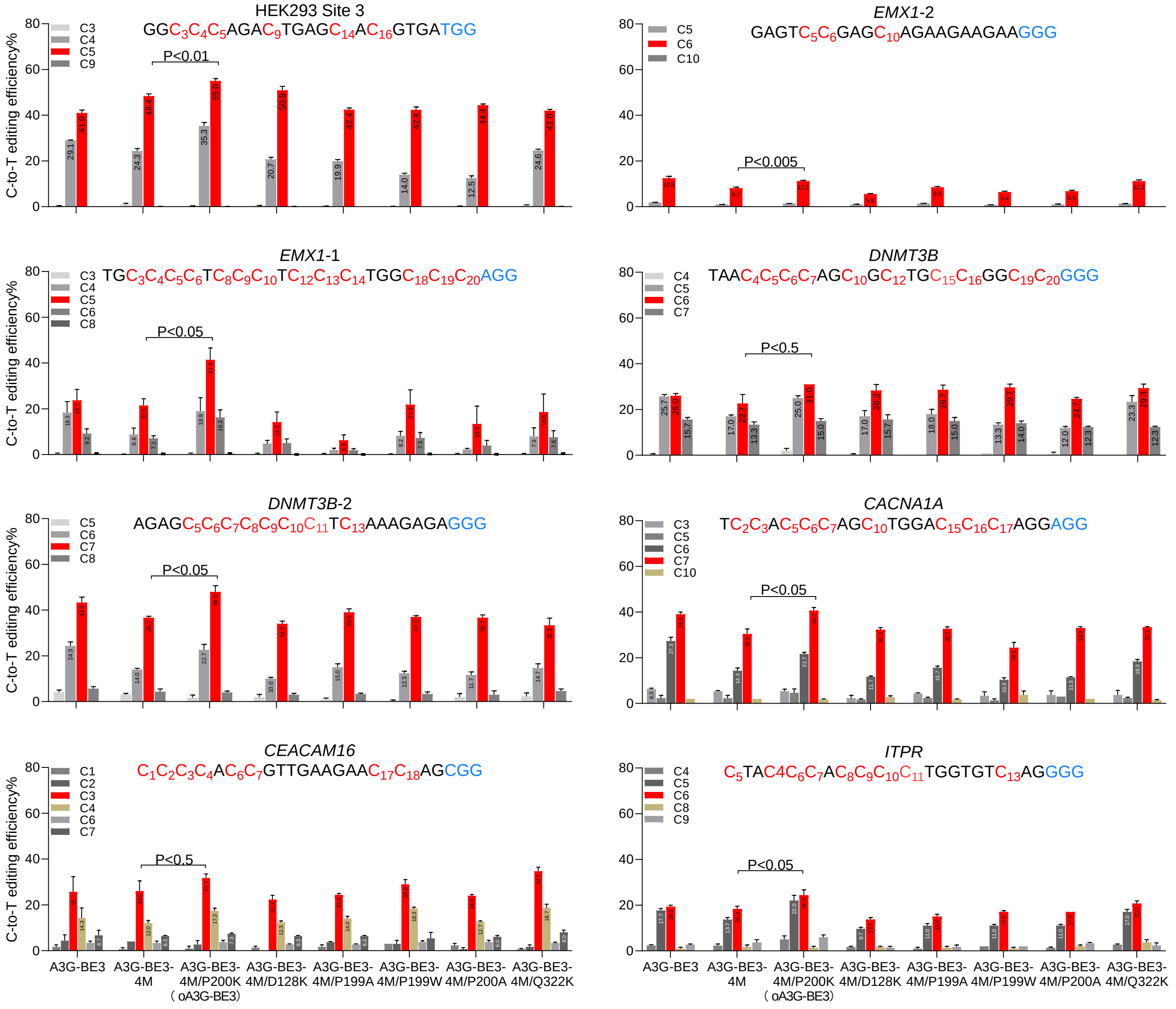

Additional file 1: Fig. S1. oA3G-BE3 outperforms the A3G-BE3 derivatives bearing different A3G mutations. The experiment was performed as in Fig. 1b. oA3G-BE3 and other A3G-BE3 derivatives are compared at a total of 8 sites, comprising the 3 sites (HEK293 site 3, *EMX1*-1 and *EMX1*-2) used in Fig. 1b and 5 additional sites (*DNMT3B,DNMT3B*-2,*CACNA1A,CEACAM16, ITPR*), where editing was quantified by NGS and Sanger sequencing, respectively.
