# Supplementary material for: Harnessing A3G for efficient and selective C-to-T conversion at C-rich sequences
Source: BMC Biol. 2021 Feb 18;19:34. doi: 10.1186/s12915-020-00879-0 (PMC7893952; doi:10.1186/s12915-020-00879-0)
Supplement: Supplementary file 2 — Additional file 2: Fig. S2. Effects of transfection condition on editing by BE3-editors. [file 12915_2020_879_MOESM2_ESM.pdf]

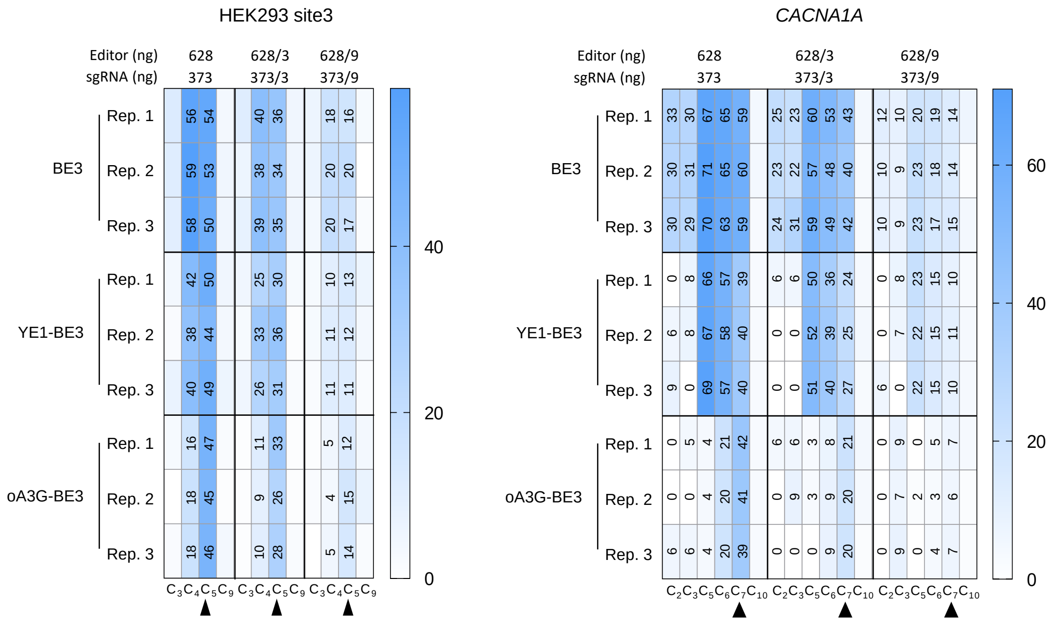

Additional file 2: Fig. S2. Effects of transfection condition on editing by BE3-editors. HEK293T cells were transfected with decreasing amounts of plasmids expressing the editors and gRNA-puromycin resistance gene, the total amount of transfected plasmids kept constant by supplementation with the respective vector backbones. Editing rates were measured by Sanger sequencing at HEK293 site 3 and *CACNA1A.*
