# Supplementary material for: Harnessing A3G for efficient and selective C-to-T conversion at C-rich sequences
Source: BMC Biol. 2021 Feb 18;19:34. doi: 10.1186/s12915-020-00879-0 (PMC7893952; doi:10.1186/s12915-020-00879-0)
Supplement: Supplementary file 3 — Additional file 3: Fig. S3. Editor performance in U2OS cells. [file 12915_2020_879_MOESM3_ESM.pdf]

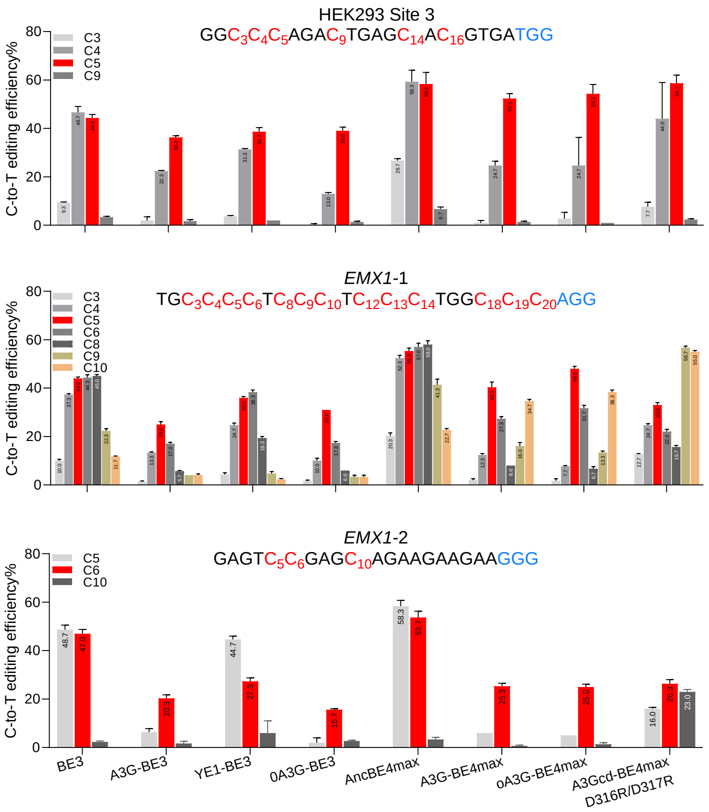

Additional file 3: Fig. S3. Editor performance in U2OS cells. The 8 editors depicted in Fig. 1a were compared at the 3 sites used in Fig. 1b in U2OS cells. The results are similar to those obtained in HEK293T cells.
