# Supplementary material for: Harnessing A3G for efficient and selective C-to-T conversion at C-rich sequences
Source: BMC Biol. 2021 Feb 18;19:34. doi: 10.1186/s12915-020-00879-0 (PMC7893952; doi:10.1186/s12915-020-00879-0)
Supplement: Supplementary file 4 — Additional file 4: Fig. S4. Sanger chromatograms for off-target editing. [file 12915_2020_879_MOESM4_ESM.pdf]

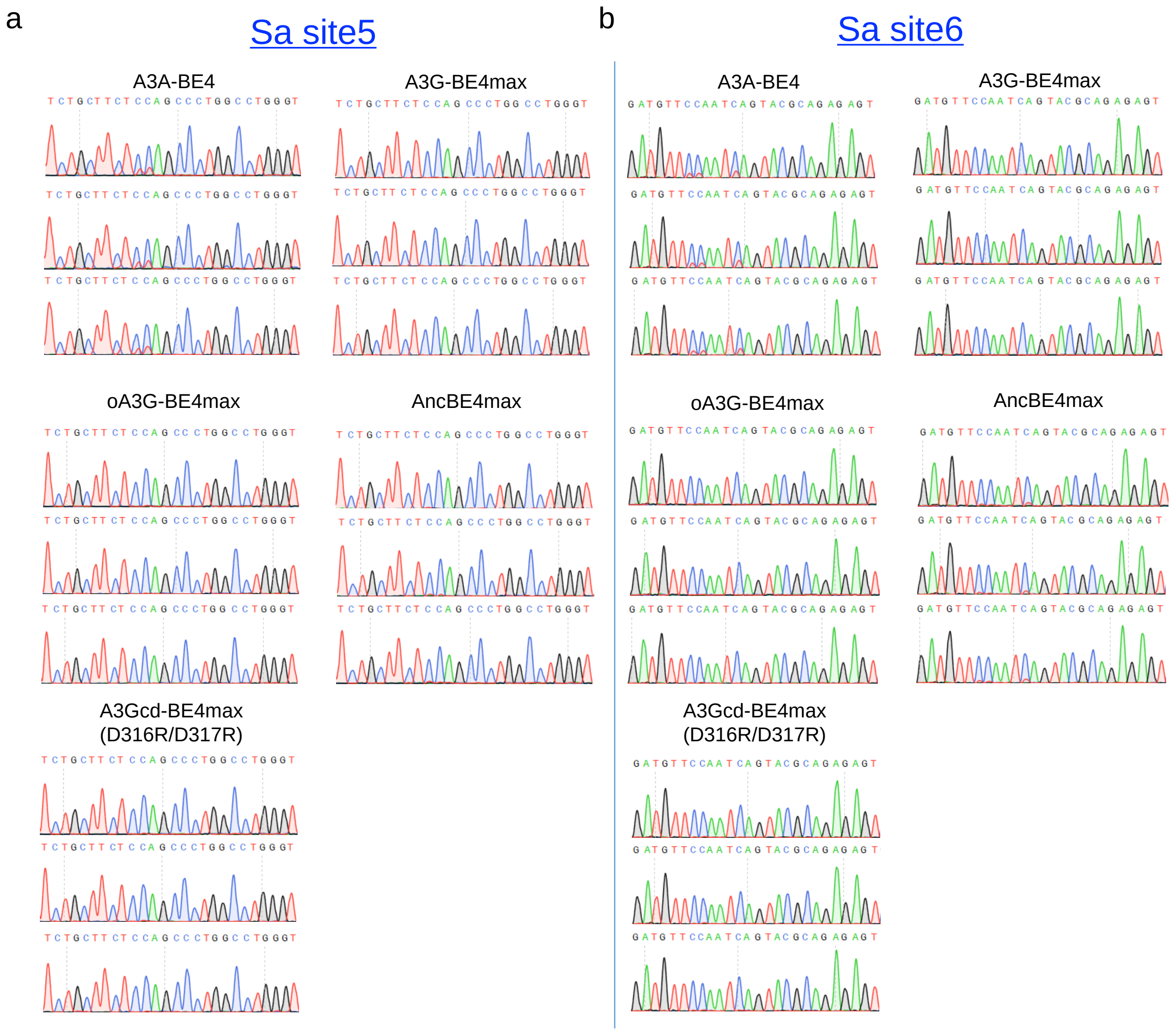

Additional file 4: Fig. S4. Sanger chromatograms for off-target editing. The experiment was done in triplicates. The sequencing traces are quantified using Editor R and presented in Fig. 4.
