# Supplementary material for: Harnessing A3G for efficient and selective C-to-T conversion at C-rich sequences
Source: BMC Biol. 2021 Feb 18;19:34. doi: 10.1186/s12915-020-00879-0 (PMC7893952; doi:10.1186/s12915-020-00879-0)
Supplement: Supplementary file 5 — Additional file 5: Fig. S5. Potential mechanisms of action of the point mutations in oA3G. [file 12915_2020_879_MOESM5_ESM.pdf]

a

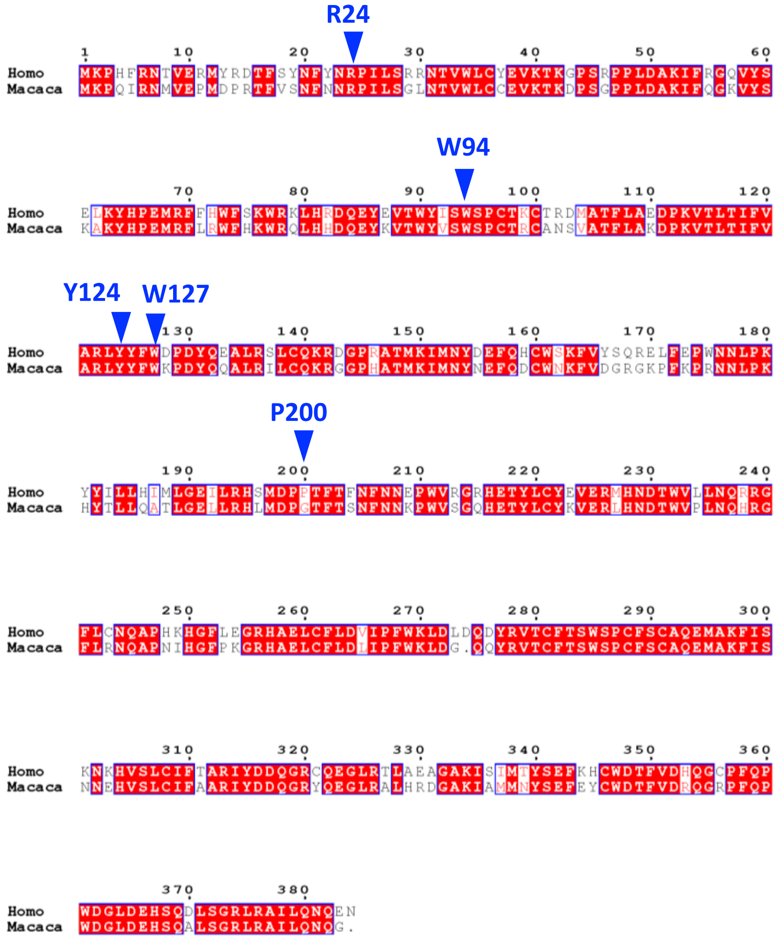

b

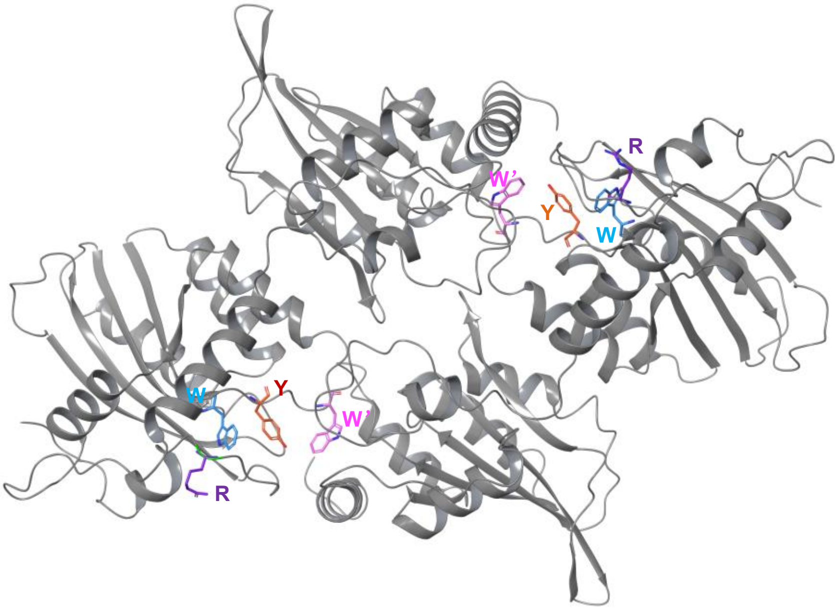

Additional file 5: Fig. S5. Potential mechanisms of action of the point mutations in oA3G. a. Sequence alignment between the human and rhesus macaque A3G proteins. Red background and red text indicate identical and strongly similar residues, respectively. The alignment was generated using CLUSTALW (https://www.genome.jp/tools-bin/clustalw) , and the graphic prepared on the ESPript 3.0 server (http://espript.ibcp.fr/ESPript/cgi-bin/ESPript.cgi). The 5 mutated residues are indicated. b. Crystal structure of dimeric rA3G (PDB: 6P40). The 4 conserved residues mutated in oA3G are highlighted. R, W, Y and W' are R24, W94, Y124 and W127, respectively.
