# Supplementary material for: Harnessing A3G for efficient and selective C-to-T conversion at C-rich sequences
Source: BMC Biol. 2021 Feb 18;19:34. doi: 10.1186/s12915-020-00879-0 (PMC7893952; doi:10.1186/s12915-020-00879-0)
Supplement: Supplementary file 6 — Additional file 6. 1. sgRNA expression vectors. 2. Base editor expression vectors. 3. Primers used for detecting editing at the genomic DNA. [file 12915_2020_879_MOESM6_ESM.docx]

**Plasmids, primers, PCR conditions**

**1. sgRNA expression vectors**

Oligos listed in the table below were annealed and inserted into BsaI-linearized vectors specified

| Name |  | Sequence | Vector |
| --- | --- | --- | --- |
| sgHEK293-Site3 up |  | ACCGGGCCCAGACTGAGCACGTGA | pGL3-U6-sgRNA-EGFP (addgene, #107721) |
| sgHEK293-Site3 down |  | AAACTCACGTGCTCAGTCTGGGCC |  |
| sg*EMX1*-sg1 up |  | ACCGTGCCCCTCCCTCCCTGGCCC |  |
| sg*EMX1*-sg1 down |  | AAACGGGCCAGGGAGGGAGGGGCA |  |
| sg*EMX1*-sg2 up |  | ACCGGAGTCCGAGCAGAAGAAGAA | pGL3-U6-sgRNA-puromycine (addgene, #51133) |
| sg*EMX1*-sg2 down |  | AAACTTCTTCTTCTGCTCGGACTC |  |
| sg*DNMT3B*-sg1 up |  | ACCGTAACCCCAGCGCTGCCGGCC |  |
| sg*DNMT3B*-sg1 down |  | AAACGGCCGGCAGCGCTGGGGTTA |  |
| sg*DNMT3B*-Site2-up |  | ACCGAGAGCCCCCCCTCAAAGAGA |  |
| sg*DNMT3B*-Site2-down |  | AAACTCTCTTTGAGGGGGGGCTCT |  |
| sg*CACNA1A*-up |  | ACCGTCCACCCAGCTGGACCCAGG |  |
| sg*CACNA1A*-down |  | AAACCCTGGGTCCAGCTGGGTGGA |  |
| sg*ITPR1*-up |  | ACCGCTACCCACCCCTGGTGTCAG |  |
| sg*ITPR1*-down |  | AAACCTGACACCAGGGGTGGGTAG |  |
| sg*CEACEAM16*-up |  | ACCGCCCCACCGTTGAAGAACCAG |  |
| sg*CEACEAM16*-down |  | AAACCTGGTTCTTCAACGGTGGGG |  |
| Sa site5-up |  | ACCGTCTGCTTCTCCAGCCCTGGC | pGL3-U6-sgRNA- BFP (addgene, #107722). |
| Sa site5-down |  | AAACGCCAGGGCTGGAGAAGCAGA |  |
| Sa site6-up |  | ACCGGATGTTCCAATCAGTACGCA |  |
| Sa site6-down |  | AAACTGCGTACTGATTGGAACATC |  |

**2. Base editor expression vectors**

To generate oA3G-BE3, we introduced R24A/ W94L/Y124/W127L into A3G-BE3 (addgene, #113415) via PCR using Phanta® Max Super-Fidelity DNA Polymerase (Vazyme) . To generate oA3G-BE4max, we amplified the oA3G coding sequence and the plasmid backbone containing BE4max coding sequence using as templates oA3G-BE3 and AncBE4max (addgene, #212164), respectively. The two amplicons were then recombined into oA3G-BE4max using ClonExpress II One Step Cloning Kit (Vazyme). The sequences of the key editors are listed below, where NLS/BPNLS, A3G/oA3G, nCas9(D10A), UGI and linker (xten, 32aa linker or GS linker) are coded in red, blue, green, orange and black, respectively, and the initiation and stop codon in bold.

**A3G-BE3:**

**ATG**AAGCCTCACTTCAGAAACACAGTGGAGCGAATGTATCGAGACACATTCTCCTACAACTTTTATAATAGACCCATCCTTTCTCGTCGGAATACCGTCTGGCTGTGCTACGAAGTGAAAACAAAGGGTCCCTCAAGGCCCCCTTTGGACGCAAAGATCTTTCGAGGCCAGGTGTATTCCGAACTTAAGTACCACCCAGAGATGAGATTCTTCCACTGGTTCAGCAAGTGGAGGAAGCTGCATCGTGACCAGGAGTATGAGGTCACCTGGTACATATCCTGGAGCCCCTGCACAAAGTGTACAAGGGATATGGCCACGTTCCTGGCCGAGGACCCGAAGGTTACCCTGACCATCTTTGTTGCCCGCCTCTACTACTTCTGGGACCCAGATTACCAGGAGGCGCTTCGCAGCCTGTGTCAGAAAAGAGACGGTCCGCGTGCCACCATGAAGATCATGAATTATGACGAATTTCAGCACTGTTGGAGCAAGTTCGTGTACAGCCAAAGAGAGCTATTTGAGCCTTGGAATAATCTGCCTAAATATTATATATTACTGCACATCATGCTGGGGGAGATTCTCAGACACTCGATGGATCCACCCACATTCACTTTCAACTTTAACAATGAACCTTGGGTCAGAGGACGGCATGAGACTTACCTGTGTTATGAGGTGGAGCGCATGCACAATGACACCTGGGTCCTGCTGAACCAGCGCAGGGGCTTTCTATGCAACCAGGCTCCACATAAACACGGTTTCCTTGAAGGCCGCCATGCAGAGCTGTGCTTCCTGGACGTGATTCCCTTTTGGAAGCTGGACCTGGACCAGGACTACAGGGTTACCTGCTTCACCTCCTGGAGCCCCTGCTTCAGCTGTGCCCAGGAAATGGCTAAATTCATTTCAAAAAACAAACACGTGAGCCTGTGCATCTTCACTGCCCGCATCTATGATGATCAAGGAAGATGTCAGGAGGGGCTGCGCACCCTGGCCGAGGCTGGGGCCAAAATTTCAATAATGACATACAGTGAATTTAAGCACTGCTGGGACACCTTTGTGGACCACCAGGGATGTCCCTTCCAGCCCTGGGATGGACTAGATGAGCACAGCCAAGACCTGAGTGGGAGGCTGCGGGCCATTCTCCAGAATCAGGAAAACAGCGGCAGCGAGACTCCCGGGACCTCAGAGTCCGCCACACCCGAAAGTGATAAAAAGTATTCTATTGGTTTAGCCATCGGCACTAATTCCGTTGGATGGGCTGTCATAACCGATGAATACAAAGTACCTTCAAAGAAATTTAAGGTGTTGGGGAACACAGACCGTCATTCGATTAAAAAGAATCTTATCGGTGCCCTCCTATTCGATAGTGGCGAAACGGCAGAGGCGACTCGCCTGAAACGAACCGCTCGGAGAAGGTATACACGTCGCAAGAACCGAATATGTTACTTACAAGAAATTTTTAGCAATGAGATGGCCAAAGTTGACGATTCTTTCTTTCACCGTTTGGAAGAGTCCTTCCTTGTCGAAGAGGACAAGAAACATGAACGGCACCCCATCTTTGGAAACATAGTAGATGAGGTGGCATATCATGAAAAGTACCCAACGATTTATCACCTCAGAAAAAAGCTAGTTGACTCAACTGATAAAGCGGACCTGAGGTTAATCTACTTGGCTCTTGCCCATATGATAAAGTTCCGTGGGCACTTTCTCATTGAGGGTGATCTAAATCCGGACAACTCGGATGTCGACAAACTGTTCATCCAGTTAGTACAAACCTATAATCAGTTGTTTGAAGAGAACCCTATAAATGCAAGTGGCGTGGATGCGAAGGCTATTCTTAGCGCCCGCCTCTCTAAATCCCGACGGCTAGAAAACCTGATCGCACAATTACCCGGAGAGAAGAAAAATGGGTTGTTCGGTAACCTTATAGCGCTCTCACTAGGCCTGACACCAAATTTTAAGTCGAACTTCGACTTAGCTGAAGATGCCAAATTGCAGCTTAGTAAGGACACGTACGATGACGATCTCGACAATCTACTGGCACAAATTGGAGATCAGTATGCGGACTTATTTTTGGCTGCCAAAAACCTTAGCGATGCAATCCTCCTATCTGACATACTGAGAGTTAATACTGAGATTACCAAGGCGCCGTTATCCGCTTCAATGATCAAAAGGTACGATGAACATCACCAAGACTTGACACTTCTCAAGGCCCTAGTCCGTCAGCAACTGCCTGAGAAATATAAGGAAATATTCTTTGATCAGTCGAAAAACGGGTACGCAGGTTATATTGACGGCGGAGCGAGTCAAGAGGAATTCTACAAGTTTATCAAACCCATATTAGAGAAGATGGATGGGACGGAAGAGTTGCTTGTAAAACTCAATCGCGAAGATCTACTGCGAAAGCAGCGGACTTTCGACAACGGTAGCATTCCACATCAAATCCACTTAGGCGAATTGCATGCTATACTTAGAAGGCAGGAGGATTTTTATCCGTTCCTCAAAGACAATCGTGAAAAGATTGAGAAAATCCTAACCTTTCGCATACCTTACTATGTGGGACCCCTGGCCCGAGGGAACTCTCGGTTCGCATGGATGACAAGAAAGTCCGAAGAAACGATTACTCCATGGAATTTTGAGGAAGTTGTCGATAAAGGTGCGTCAGCTCAATCGTTCATCGAGAGGATGACCAACTTTGACAAGAATTTACCGAACGAAAAAGTATTGCCTAAGCACAGTTTACTTTACGAGTATTTCACAGTGTACAATGAACTCACGAAAGTTAAGTATGTCACTGAGGGCATGCGTAAACCCGCCTTTCTAAGCGGAGAACAGAAGAAAGCAATAGTAGATCTGTTATTCAAGACCAACCGCAAAGTGACAGTTAAGCAATTGAAAGAGGACTACTTTAAGAAAATTGAATGCTTCGATTCTGTCGAGATCTCCGGGGTAGAAGATCGATTTAATGCGTCACTTGGTACGTATCATGACCTCCTAAAGATAATTAAAGATAAGGACTTCCTGGATAACGAAGAGAATGAAGATATCTTAGAAGATATAGTGTTGACTCTTACCCTCTTTGAAGATCGGGAAATGATTGAGGAAAGACTAAAAACATACGCTCACCTGTTCGACGATAAGGTTATGAAACAGTTAAAGAGGCGTCGCTATACGGGCTGGGGACGATTGTCGCGGAAACTTATCAACGGGATAAGAGACAAGCAAAGTGGTAAAACTATTCTCGATTTTCTAAAGAGCGACGGCTTCGCCAATAGGAACTTTATGCAGCTGATCCATGATGACTCTTTAACCTTCAAAGAGGATATACAAAAGGCACAGGTTTCCGGACAAGGGGACTCATTGCACGAACATATTGCGAATCTTGCTGGTTCGCCAGCCATCAAAAAGGGCATACTCCAGACAGTCAAAGTAGTGGATGAGCTAGTTAAGGTCATGGGACGTCACAAACCGGAAAACATTGTAATCGAGATGGCACGCGAAAATCAAACGACTCAGAAGGGGCAAAAAAACAGTCGAGAGCGGATGAAGAGAATAGAAGAGGGTATTAAAGAACTGGGCAGCCAGATCTTAAAGGAGCATCCTGTGGAAAATACCCAATTGCAGAACGAGAAACTTTACCTCTATTACCTACAAAATGGAAGGGACATGTATGTTGATCAGGAACTGGACATAAACCGTTTATCTGATTACGACGTCGATCACATTGTACCCCAATCCTTTTTGAAGGACGATTCAATCGACAATAAAGTGCTTACACGCTCGGATAAGAACCGAGGGAAAAGTGACAATGTTCCAAGCGAGGAAGTCGTAAAGAAAATGAAGAACTATTGGCGGCAGCTCCTAAATGCGAAACTGATAACGCAAAGAAAGTTCGATAACTTAACTAAAGCTGAGAGGGGTGGCTTGTCTGAACTTGACAAGGCCGGATTTATTAAACGTCAGCTCGTGGAAACCCGCCAAATCACAAAGCATGTTGCACAGATACTAGATTCCCGAATGAATACGAAATACGACGAGAACGATAAGCTGATTCGGGAAGTCAAAGTAATCACTTTAAAGTCAAAATTGGTGTCGGACTTCAGAAAGGATTTTCAATTCTATAAAGTTAGGGAGATAAATAACTACCACCATGCGCACGACGCTTATCTTAATGCCGTCGTAGGGACCGCACTCATTAAGAAATACCCGAAGCTAGAAAGTGAGTTTGTGTATGGTGATTACAAAGTTTATGACGTCCGTAAGATGATCGCGAAAAGCGAACAGGAGATAGGCAAGGCTACAGCCAAATACTTCTTTTATTCTAACATTATGAATTTCTTTAAGACGGAAATCACTCTGGCAAACGGAGAGATACGCAAACGACCTTTAATTGAAACCAATGGGGAGACAGGTGAAATCGTATGGGATAAGGGCCGGGACTTCGCGACGGTGAGAAAAGTTTTGTCCATGCCCCAAGTCAACATAGTAAAGAAAACTGAGGTGCAGACCGGAGGGTTTTCAAAGGAATCGATTCTTCCAAAAAGGAATAGTGATAAGCTCATCGCTCGTAAAAAGGACTGGGACCCGAAAAAGTACGGTGGCTTCGATAGCCCTACAGTTGCCTATTCTGTCCTAGTAGTGGCAAAAGTTGAGAAGGGAAAATCCAAGAAACTGAAGTCAGTCAAAGAATTATTGGGGATAACGATTATGGAGCGCTCGTCTTTTGAAAAGAACCCCATCGACTTCCTTGAGGCGAAAGGTTACAAGGAAGTAAAAAAGGATCTCATAATTAAACTACCAAAGTATAGTCTGTTTGAGTTAGAAAATGGCCGAAAACGGATGTTGGCTAGCGCCGGAGAGCTTCAAAAGGGGAACGAACTCGCACTACCGTCTAAATACGTGAATTTCCTGTATTTAGCGTCCCATTACGAGAAGTTGAAAGGTTCACCTGAAGATAACGAACAGAAGCAACTTTTTGTTGAGCAGCACAAACATTATCTCGACGAAATCATAGAGCAAATTTCGGAATTCAGTAAGAGAGTCATCCTAGCTGATGCCAATCTGGACAAAGTATTAAGCGCATACAACAAGCACAGGGATAAACCCATACGTGAGCAGGCGGAAAATATTATCCATTTGTTTACTCTTACCAACCTCGGCGCTCCAGCCGCATTCAAGTATTTTGACACAACGATAGATCGCAAACGATACACTTCTACCAAGGAGGTGCTAGACGCGACACTGATTCACCAATCCATCACGGGATTATATGAAACTCGGATAGATTTGTCACAGCTTGGGGGTGACTCTGGTGGTTCTACTAATCTGTCAGATATTATTGAAAAGGAGACCGGTAAGCAACTGGTTATCCAGGAATCCATCCTCATGCTCCCAGAGGAGGTGGAAGAAGTCATTGGGAACAAGCCGGAAAGCGATATACTCGTGCACACCGCCTACGACGAGAGCACCGACGAGAATGTCATGCTTCTGACTAGCGACGCCCCTGAATACAAGCCTTGGGCTCTGGTCATACAGGATAGCAACGGTGAGAACAAGATTAAGATGCTCTCTGGTGGTTCTCCCAAGAAGAAGAGGAAAGTC**TAA**

**A3G-BE4max:**

**ATG**AAACGGACAGCCGACGGAAGCGAGTTCGAGTCACCAAAGAAGAAGCGGAAAGTCATGAAGCCTCACTTCAGAAACACAGTGGAGCGAATGTATCGAGACACATTCTCCTACAACTTTTATAATAGACCCATCCTTTCTCGTCGGAATACCGTCTGGCTGTGCTACGAAGTGAAAACAAAGGGTCCCTCAAGGCCCCCTTTGGACGCAAAGATCTTTCGAGGCCAGGTGTATTCCGAACTTAAGTACCACCCAGAGATGAGATTCTTCCACTGGTTCAGCAAGTGGAGGAAGCTGCATCGTGACCAGGAGTATGAGGTCACCTGGTACATATCCTGGAGCCCCTGCACAAAGTGTACAAGGGATATGGCCACGTTCCTGGCCGAGGACCCGAAGGTTACCCTGACCATCTTTGTTGCCCGCCTCTACTACTTCTGGGACCCAGATTACCAGGAGGCGCTTCGCAGCCTGTGTCAGAAAAGAGACGGTCCGCGTGCCACCATGAAGATCATGAATTATGACGAATTTCAGCACTGTTGGAGCAAGTTCGTGTACAGCCAAAGAGAGCTATTTGAGCCTTGGAATAATCTGCCTAAATATTATATATTACTGCACATCATGCTGGGGGAGATTCTCAGACACTCGATGGATCCACCCACATTCACTTTCAACTTTAACAATGAACCTTGGGTCAGAGGACGGCATGAGACTTACCTGTGTTATGAGGTGGAGCGCATGCACAATGACACCTGGGTCCTGCTGAACCAGCGCAGGGGCTTTCTATGCAACCAGGCTCCACATAAACACGGTTTCCTTGAAGGCCGCCATGCAGAGCTGTGCTTCCTGGACGTGATTCCCTTTTGGAAGCTGGACCTGGACCAGGACTACAGGGTTACCTGCTTCACCTCCTGGAGCCCCTGCTTCAGCTGTGCCCAGGAAATGGCTAAATTCATTTCAAAAAACAAACACGTGAGCCTGTGCATCTTCACTGCCCGCATCTATGATGATCAAGGAAGATGTCAGGAGGGGCTGCGCACCCTGGCCGAGGCTGGGGCCAAAATTTCAATAATGACATACAGTGAATTTAAGCACTGCTGGGACACCTTTGTGGACCACCAGGGATGTCCCTTCCAGCCCTGGGATGGACTAGATGAGCACAGCCAAGACCTGAGTGGGAGGCTGCGGGCCATTCTCCAGAATCAGGAAAACTCTGGAGGATCTAGCGGAGGATCCTCTGGCAGCGAGACACCAGGAACAAGCGAGTCAGCAACACCAGAGAGCAGTGGCGGCAGCAGCGGCGGCAGCGACAAGAAGTACAGCATCGGCCTGGCCATCGGCACCAACTCTGTGGGCTGGGCCGTGATCACCGACGAGTACAAGGTGCCCAGCAAGAAATTCAAGGTGCTGGGCAACACCGACCGGCACAGCATCAAGAAGAACCTGATCGGAGCCCTGCTGTTCGACAGCGGCGAAACAGCCGAGGCCACCCGGCTGAAGAGAACCGCCAGAAGAAGATACACCAGACGGAAGAACCGGATCTGCTATCTGCAAGAGATCTTCAGCAACGAGATGGCCAAGGTGGACGACAGCTTCTTCCACAGACTGGAAGAGTCCTTCCTGGTGGAAGAGGATAAGAAGCACGAGCGGCACCCCATCTTCGGCAACATCGTGGACGAGGTGGCCTACCACGAGAAGTACCCCACCATCTACCACCTGAGAAAGAAACTGGTGGACAGCACCGACAAGGCCGACCTGCGGCTGATCTATCTGGCCCTGGCCCACATGATCAAGTTCCGGGGCCACTTCCTGATCGAGGGCGACCTGAACCCCGACAACAGCGACGTGGACAAGCTGTTCATCCAGCTGGTGCAGACCTACAACCAGCTGTTCGAGGAAAACCCCATCAACGCCAGCGGCGTGGACGCCAAGGCCATCCTGTCTGCCAGACTGAGCAAGAGCAGACGGCTGGAAAATCTGATCGCCCAGCTGCCCGGCGAGAAGAAGAATGGCCTGTTCGGAAACCTGATTGCCCTGAGCCTGGGCCTGACCCCCAACTTCAAGAGCAACTTCGACCTGGCCGAGGATGCCAAACTGCAGCTGAGCAAGGACACCTACGACGACGACCTGGACAACCTGCTGGCCCAGATCGGCGACCAGTACGCCGACCTGTTTCTGGCCGCCAAGAACCTGTCCGACGCCATCCTGCTGAGCGACATCCTGAGAGTGAACACCGAGATCACCAAGGCCCCCCTGAGCGCCTCTATGATCAAGAGATACGACGAGCACCACCAGGACCTGACCCTGCTGAAAGCTCTCGTGCGGCAGCAGCTGCCTGAGAAGTACAAAGAGATTTTCTTCGACCAGAGCAAGAACGGCTACGCCGGCTACATTGACGGCGGAGCCAGCCAGGAAGAGTTCTACAAGTTCATCAAGCCCATCCTGGAAAAGATGGACGGCACCGAGGAACTGCTCGTGAAGCTGAACAGAGAGGACCTGCTGCGGAAGCAGCGGACCTTCGACAACGGCAGCATCCCCCACCAGATCCACCTGGGAGAGCTGCACGCCATTCTGCGGCGGCAGGAAGATTTTTACCCATTCCTGAAGGACAACCGGGAAAAGATCGAGAAGATCCTGACCTTCCGCATCCCCTACTACGTGGGCCCTCTGGCCAGGGGAAACAGCAGATTCGCCTGGATGACCAGAAAGAGCGAGGAAACCATCACCCCCTGGAACTTCGAGGAAGTGGTGGACAAGGGCGCTTCCGCCCAGAGCTTCATCGAGCGGATGACCAACTTCGATAAGAACCTGCCCAACGAGAAGGTGCTGCCCAAGCACAGCCTGCTGTACGAGTACTTCACCGTGTATAACGAGCTGACCAAAGTGAAATACGTGACCGAGGGAATGAGAAAGCCCGCCTTCCTGAGCGGCGAGCAGAAAAAGGCCATCGTGGACCTGCTGTTCAAGACCAACCGGAAAGTGACCGTGAAGCAGCTGAAAGAGGACTACTTCAAGAAAATCGAGTGCTTCGACTCCGTGGAAATCTCCGGCGTGGAAGATCGGTTCAACGCCTCCCTGGGCACATACCACGATCTGCTGAAAATTATCAAGGACAAGGACTTCCTGGACAATGAGGAAAACGAGGACATTCTGGAAGATATCGTGCTGACCCTGACACTGTTTGAGGACAGAGAGATGATCGAGGAACGGCTGAAAACCTATGCCCACCTGTTCGACGACAAAGTGATGAAGCAGCTGAAGCGGCGGAGATACACCGGCTGGGGCAGGCTGAGCCGGAAGCTGATCAACGGCATCCGGGACAAGCAGTCCGGCAAGACAATCCTGGATTTCCTGAAGTCCGACGGCTTCGCCAACAGAAACTTCATGCAGCTGATCCACGACGACAGCCTGACCTTTAAAGAGGACATCCAGAAAGCCCAGGTGTCCGGCCAGGGCGATAGCCTGCACGAGCACATTGCCAATCTGGCCGGCAGCCCCGCCATTAAGAAGGGCATCCTGCAGACAGTGAAGGTGGTGGACGAGCTCGTGAAAGTGATGGGCCGGCACAAGCCCGAGAACATCGTGATCGAAATGGCCAGAGAGAACCAGACCACCCAGAAGGGACAGAAGAACAGCCGCGAGAGAATGAAGCGGATCGAAGAGGGCATCAAAGAGCTGGGCAGCCAGATCCTGAAAGAACACCCCGTGGAAAACACCCAGCTGCAGAACGAGAAGCTGTACCTGTACTACCTGCAGAATGGGCGGGATATGTACGTGGACCAGGAACTGGACATCAACCGGCTGTCCGACTACGATGTGGACCATATCGTGCCTCAGAGCTTTCTGAAGGACGACTCCATCGACAACAAGGTGCTGACCAGAAGCGACAAGAACCGGGGCAAGAGCGACAACGTGCCCTCCGAAGAGGTCGTGAAGAAGATGAAGAACTACTGGCGGCAGCTGCTGAACGCCAAGCTGATTACCCAGAGAAAGTTCGACAATCTGACCAAGGCCGAGAGAGGCGGCCTGAGCGAACTGGATAAGGCCGGCTTCATCAAGAGACAGCTGGTGGAAACCCGGCAGATTACAAAGCACGTGGCACAGATCCTGGACTCCCGGATGAACACTAAGTACGACGAGAATGACAAGCTGATCCGGGAAGTGAAAGTGATCACCCTGAAGTCCAAGCTGGTGTCCGATTTCCGGAAGGATTTCCAGTTTTACAAAGTGCGCGAGATCAACAACTACCACCACGCCCACGACGCCTACCTAAACGCCGTCGTGGGAACCGCACTGATCAAAAAGTACCCTAAGCTGGAAAGCGAGTTCGTGTACGGCGACTACAAGGTGTACGACGTGCGGAAGATGATCGCCAAGAGCGAGCAGGAAATCGGCAAGGCTACCGCCAAGTACTTCTTCTACAGCAACATCATGAACTTTTTCAAGACCGAGATTACCCTGGCCAACGGCGAGATCCGGAAGCGGCCTCTGATCGAGACAAACGGCGAAACCGGGGAGATCGTGTGGGATAAGGGCCGGGATTTTGCCACCGTGCGGAAAGTGCTGAGCATGCCCCAAGTGAATATCGTGAAAAAGACCGAGGTGCAGACAGGCGGCTTCAGCAAAGAGTCTATCAGACCCAAGAGGAACAGCGATAAGCTGATCGCCAGAAAGAAGGACTGGGACCCTAAGAAGTACGGCGGCTTCGTGAGCCCCACCGTGGCCTATTCTGTGCTGGTGGTGGCCAAAGTGGAAAAGGGCAAGTCCAAGAAACTGAAGAGTGTGAAAGAGCTGCTGGGGATCACCATCATGGAAAGAAGCAGCTTCGAGAAGAATCCCATCGACTTTCTGGAAGCCAAGGGCTACAAAGAAGTGAAAAAGGACCTGATCATCAAGCTGCCTAAGTACTCCCTGTTCGAGCTGGAAAACGGCCGGAAGAGAATGCTGGCCTCTGCCAGATTCCTGCAGAAGGGAAACGAACTGGCCCTGCCCTCCAAATATGTGAACTTCCTGTACCTGGCCAGCCACTATGAGAAGCTGAAGGGCTCCCCCGAGGATAATGAGCAGAAACAGCTGTTTGTGGAACAGCACAAGCACTACCTGGACGAGATCATCGAGCAGATCAGCGAGTTCTCCAAGAGAGTGATCCTGGCCGACGCTAATCTGGACAAAGTGCTGTCCGCCTACAACAAGCACCGGGATAAGCCCATCAGAGAGCAGGCCGAGAATATCATCCACCTGTTTACCCTGACCAATCTGGGAGCCCCTAGAGCCTTCAAGTACTTTGACACCACCATCGACCGGAAGGTGTACAGAAGCACCAAAGAGGTGCTGGACGCCACCCTGATCCACCAGAGCATCACCGGCCTGTACGAGACACGGATCGACCTGTCTCAGCTGGGAGGTGACAGCGGCGGGAGCGGCGGGAGCGGGGGGAGCACTAATCTGAGCGACATCATTGAGAAGGAGACTGGGAAACAGCTGGTCATTCAGGAGTCCATCCTGATGCTGCCTGAGGAGGTGGAGGAAGTGATCGGCAACAAGCCAGAGTCTGACATCCTGGTGCACACCGCCTACGACGAGTCCACAGATGAGAATGTGATGCTGCTGACCTCTGACGCCCCCGAGTATAAGCCTTGGGCCCTGGTCATCCAGGATTCTAACGGCGAGAATAAGATCAAGATGCTGAGCGGAGGATCCGGAGGATCTGGAGGCAGCACCAACCTGTCTGACATCATCGAGAAGGAGACAGGCAAGCAGCTGGTCATCCAGGAGAGCATCCTGATGCTGCCCGAAGAAGTCGAAGAAGTGATCGGAAACAAGCCTGAGAGCGATATCCTGGTCCATACCGCCTACGACGAGAGTACCGACGAAAATGTGATGCTGCTGACATCCGACGCCCCAGAGTATAAGCCCTGGGCTCTGGTCATCCAGGATTCCAACGGAGAGAACAAAATCAAAATGCTGTCTGGCGGCTCAAAAAGAACCGCCGACGGCAGCGAATTCGAGCCCAAGAAGAAGAGGAAAGTC**TAA**

**oA3G:**

**The editor oA3G-BE3 and oA3G-BE4max construct is based on A3G-BE3 and A3G-BE4max by introduced mutant to A3G. The DNA sequencing is listed below, and the mutant base is in bold while the mutant amino acid sequence is underlined.**

**ATG**AAGCCTCACTTCAGAAACACAGTGGAGCGAATGTATCGAGACACATTCTCCTACAACTTTTATAAT**GC**ACCCATCCTTTCTCGTCGGAATACCGTCTGGCTGTGCTACGAAGTGAAAACAAAGGGTCCCTCAAGGCCCCCTTTGGACGCAAAGATCTTTCGAGGCCAGGTGTATTCCGAACTTAAGTACCACCCAGAGATGAGATTCTTCCACTGGTTCAGCAAGTGGAGGAAGCTGCATCGTGACCAGGAGTATGAGGTCACCTGGTACATATCCT**T**GAGCCCCTGCACAAAGTGTACAAGGGATATGGCCACGTTCCTGGCCGAGGACCCGAAGGTTACCCTGACCATCTTTGTTGCCCGCCTC**GC**CTACTTC**CTT**GACCCAGATTACCAGGAGGCGCTTCGCAGCCTGTGTCAGAAAAGAGACGGTCCGCGTGCCACCATGAAGATCATGAATTATGACGAATTTCAGCACTGTTGGAGCAAGTTCGTGTACAGCCAAAGAGAGCTATTTGAGCCTTGGAATAATCTGCCTAAATATTATATATTACTGCACATCATGCTGGGGGAGATTCTCAGACACTCGATGGATCCA**AAG**ACATTCACTTTCAACTTTAACAATGAACCTTGGGTCAGAGGACGGCATGAGACTTACCTGTGTTATGAGGTGGAGCGCATGCACAATGACACCTGGGTCCTGCTGAACCAGCGCAGGGGCTTTCTATGCAACCAGGCTCCACATAAACACGGTTTCCTTGAAGGCCGCCATGCAGAGCTGTGCTTCCTGGACGTGATTCCCTTTTGGAAGCTGGACCTGGACCAGGACTACAGGGTTACCTGCTTCACCTCCTGGAGCCCCTGCTTCAGCTGTGCCCAGGAAATGGCTAAATTCATTTCAAAAAACAAACACGTGAGCCTGTGCATCTTCACTGCCCGCATCTATGATGATCAAGGAAGATGTCAGGAGGGGCTGCGCACCCTGGCCGAGGCTGGGGCCAAAATTTCAATAATGACATACAGTGAATTTAAGCACTGCTGGGACACCTTTGTGGACCACCAGGGATGTCCCTTCCAGCCCTGGGATGGACTAGATGAGCACAGCCAAGACCTGAGTGGGAGGCTGCGGGCCATTCTCCAGAATCAGGAAAAC

**3. Primers used for detecting editing at the genomic DNA**

For the detection using Sanger sequencing, the target sites were amplified with the primers listed below, and then sequenced using one of these primers. For detection by deep–sequencing, the same target regions were amplified, but with Illumina adaptors and sample barcodes appended.

| Primer name | Primer sequence |
| --- | --- |
| sgHEK293-Site3-F | GGGAAACGCCCATGCAATTAG |
| sgHEK293-Site3-BAR16-F1 | CCGTCCATGGGAAACGCCCATGCAATTAG |
| sgHEK293-Site3-BAR17-F2 | GTAGAGCGGGAAACGCCCATGCAATTAG |
| sgHEK293-Site3-BAR18-F3 | GTCCGCAGGGAAACGCCCATGCAATTAG |
| sgHEK293-Site3-BAR19-F4 | GTGAAAGGGAAACGCCCATGCAATTAG |
| sgHEK293-Site3-BAR5-F5 | ACAGTGGATGGGGAAACGCCCATGCAATTAG |
| sgHEK293-Site3-BAR6-F6 | GCCAATGGGAAACGCCCATGCAATTAG |
| sgHEK293-Site3-BAR7-F7 | CAGATCAGGGAAACGCCCATGCAATTAG |
| sgHEK293-Site3-BAR8-F8 | ACTTGATGGGGAAACGCCCATGCAATTAG |
| sgHEK293-Site3-BAR9-F9 | GATCAGCACGGGAAACGCCCATGCAATTAG |
| sgHEK293-Site3-R | CTGCACCGGGATACTGGTTGAC |
| sg*EMX1*-sg1,2-F | AGCCACAGTGTCTCCGAGG |
| sg*EMX1*-sg1,2-BAR8-F1 | ACTTGATGGGGGCCCCTAACCCTATGTAGC |
| sg*EMX1*-sg1,2-BAR9-F2 | GATCAGCACGGGGCCCCTAACCCTATGTAGC |
| sg*EMX1*-sg1,2-BAR10-F3 | TAGCTTGTGCGGGGCCCCTAACCCTATGTAGC |
| sg*EMX1*-sg1,2-BAR11-F4 | GGCTACGGGGCCCCTAACCCTATGTAGC |
| sg*EMX1*-sg1,2-BAR20-F5 | GTGGCCTGGGGCCCCTAACCCTATGTAGC |
| sg*EMX1*-sg1,2-BAR21-F6 | GTTTCGGGGGCCCCTAACCCTATGTAGC |
| sg*EMX1*-sg1,2-BAR22-F7 | CGTACGGGGGGCCCCTAACCCTATGTAGC |
| sg*EMX1*-sg1,2-BAR23-F8 | GAGTGGAGGGGGCCCCTAACCCTATGTAGC |
| sg*EMX1*-sg1,2-BAR24-F9 | GGTAGCAGGGGCCCCTAACCCTATGTAGC |
| sg*EMX1*-sg1,2-R | CCCCGGTGTGTAGCTCAGC |
| sg*DNMT3B*-sg1-F | GCTTCCTCGCAGCAGCTGCTC |
| sg*DNMT3B*-sg1-R | GGCTGGCTGGCGGGAGGC |
| sg*DNMT3B*-Site2-F | TTTAGCAGCTGGTGTCAGGGC |
| sg*DNMT3B*-Site2-R | GGGCTTCACTGAGTCTCCAC |
| sg*CACNA1A*-F | CCCTTGTCCACACACTGCTCTC |
| sg*CACNA1A*-R | GCTTTCGTGAGCCATCCTGC |
| sg*ITPR1*-F | AATGCAACTAGGTTAGGTGCATC |
| sg*ITPR1*-R | TGACCTGAGCTACCTGTTTG |
| sg*CEACEAM16*-F | AAGGCCCTCAGAGCAGGTGG |
| sg*CEACEAM16*-R | CTCACACTGATAGGCGCCGG |
| Sa site5-F | ATGTGGGCTGCCTAGAAAGG |
| Sa site5-R | CCCAGCCAAACTTGTCAACC |
| Sa site6-F | TAGGATGCCCTACATCTGCTCTC |
| Sa site6-R | CGGAAAAGCGATCCAGGTGC |

PCR Condition (except Sa site5)

94℃ 5min

94℃ 30s

68℃(-1℃/cycle) 30s x 10 cycles

72℃ 30s

94℃ 30s

58℃ 30s x 25cycles

72℃ 30s

72℃ 7min

4℃ ∞

Sa site5:

94℃ 5min

94℃ 30s

55℃ 30s x 32cycles

72℃ 30s

72℃ 7min

4℃ ∞
